# The effects of video observation of chewing during lunch on masticatory ability, food intake, cognition, activities of daily living, depression, and quality of life in older adults with dementia: a study protocol of an adjusted randomized controlled trial

**DOI:** 10.1186/s12877-016-0205-6

**Published:** 2016-02-04

**Authors:** Johanna G. Douma, Karin M. Volkers, Pieter Jelle Vuijk, Erik J. A. Scherder

**Affiliations:** Department of Clinical Neuropsychology, VU University, Van der Boechorststraat 1, 1081 BT Amsterdam, The Netherlands; University of Groningen, Center for Human Movement Sciences, Antonius Deusinglaan 1, 9713 AV Groningen, The Netherlands

**Keywords:** Mastication, Older adults, Dementia, Cognition, Food intake, Activities of daily living, Depression, Quality of life, Mirror neuron system, Action observation

## Abstract

**Background:**

Masticatory functioning alters with age. However, mastication has been found to be related to, for example, cognitive functioning, food intake, and some aspects of activities of daily living. Since cognitive functioning and activities of daily living show a decline in older adults with dementia, improving masticatory functioning may be of relevance to them. A possible way to improve mastication may be showing videos of people who are chewing. Observing chewing movements may activate the mirror neuron system, which becomes also activated during the execution of that same movement. The primary hypothesis is that the observation of chewing has a beneficial effect on masticatory functioning, or, more specifically, masticatory ability of older adults with dementia. Secondary, the intervention is hypothesized to have beneficial effects on food intake, cognition, activities of daily living, depression, and quality of life.

**Methods/Design:**

An adjusted parallel randomized controlled trial is being performed in dining rooms of residential care settings. Older adults with dementia, for whom also additional eligibility criteria apply, are randomly assigned to the experimental (videos of chewing people) or control condition (videos of nature and buildings), by drawing folded pieces of paper. Participants who are able to watch each other’s videos are assigned to the same study condition. The intervention takes place during lunchtime, from Monday to Friday, for 3 months. During four moments of measurement, masticatory ability, food intake, cognitive functioning, activities of daily living, depression, and quality of life are assessed. Tests administrators blind to the group allocation administer the tests to participants.

**Discussion:**

The goal of this study is to examine the effects of video observation of chewing on masticatory ability and several secondary outcome measures. In this study, the observation of chewing is added to the execution of the same action (i.e., during eating). Beneficial effects on masticatory ability, and consequently on the other outcome measures are hypothesized. The intervention may be easily integrated into daily care, and might add to the lives of the increasing number of older adults with dementia by beneficially influencing multiple daily life functions.

**Trial registration:**

NTR5124. Registration date: 30 March 2015.

## Background

The number of people with dementia worldwide is increasing at a high rate and is expected to increase even more in the coming decades [1]. By far most of the people with dementia are aged adults [2]. In the types of dementia that are most common (i.e., Alzheimer’s disease and vascular dementia), brain areas such as the hippocampus and the frontal lobe are affected [3]. The hippocampus is an area of great importance for memory functioning, and part of the frontal lobe, the prefrontal cortex, plays an important role in both executive functions and memory [4]. Indeed, both executive functions and memory show a decline in dementia [2].

Besides the decline in cognition in dementia, a function that alters in ageing in general is masticatory functioning [5]. This is the case for both objective and subjective measures of mastication [5]. With regard to the objective measures, with ageing for example the number of teeth declines [6], there are changes with regard to swallowing [7], and the mixing ability decreases from young to old age [8]. It is of note that masticatory performance was no longer significantly related with age when only older adults were included and other variables were taken into account (i.e., loss of teeth, occlusal force, salivary flow, and gender) [9]. In addition to the objective changes, subjectively, people report a lower chewing ability with age. That is, a study with participants aged 50 and above found that, on average, chewing ability significantly decreased over a period of 7 years [10]. The significant decrease was not found between baseline and a follow-up after 3 years, but was found between the follow-up at 3 years and 7 years. Additionally, even though chewing difficulty may not be significantly related to dementia [11], it appears to be significantly related to cognitive impairment [12, 13].

Chewing has been found to have beneficial effects on physiological functions, which may in turn positively influence cognitive functioning. That is, chewing has been found to cause an increase in heart rate [14], and, if the chewing movement is of at least moderate intensity, also a significant increase in the cerebral blood flow [15]. The latter increase has been found by measuring the velocity of the blood flow through the middle cerebral artery, during chewing movements of moderate to high intensity [15]. Chewing gum may lead to activation in for example, the right prefrontal cortex; an activation that was even larger in older adults than in younger people [16]. Stimulation of brain areas, such as the prefrontal area, by means of chewing could have a beneficial effect on cognitive functions such as executive functions. A beneficial effect on cognitive functioning could possibly be enhanced by an increase in glucose [17]. A recent review showed that mastication is indeed related to cognition [5]. Even in younger adults, chewing gum has been shown to improve cognitive task performance for cognitive domains such as short-term memory [17, 18], long-term memory [17], and the executive function working memory [18]. However, beneficial effects on these functions are not found for all tests [18] or in all studies [19, 20], and are also not necessarily generalizable to all cognitive domains [21]. For example, one study found no significant effects of chewing gum on spatial task performance [21]. With regard to attention, various findings are reported in different studies [17, 18, 20, 22]. Because of the limited number of studies, and the different findings between these studies, more research into the causal effects of chewing on cognition and specific cognitive domains remains necessary, especially in older adults (see also [5]).

Beside the possible beneficial effects of mastication on cognition, mastication also seems to be related to food intake [23]. Most of the described studies that focused on the relation of mastication to food or nutrient intake, found that better mastication was related to higher intake of, for example, vegetables or vitamin A. In addition, chewing also seems to be related to several other aspects of daily life. For example, a relation between masticatory ability and most measured aspects of activities of daily living (ADL) was found [24, 25], and also between chewing ability and depression [24]. As for older adults’ quality of life (QoL), a relation between masticatory ability and QoL has been found in one [26], but not in another study [24]. The two studies differed in their ways of assessing masticatory ability (i.e., number of foods that a participant was able to chew [26] versus the score on a colour-changing chewing gum test [24]), and used different questionnaires/scales to measure QoL. Additionally, the studies grouped participants differently based on their score on masticatory ability. Even though the results are inconsistent, a beneficial effect of chewing on QoL may be expected, since physical and cognitive functioning are seen as a part of QoL, or as factors that influence this concept [27].

Overall, mastication may be positively related to cognition, food intake, ADL, depression, and QoL. It has been found that almost a third of the people with dementia living in residential care settings have low food intake [28]. In addition, cognitive functions [2] and ADL [29] show a decline in older adults with dementia. Therefore, it is of clinical relevance to develop an intervention that might improve masticatory function, without increasing the already high burden of the nursing staff. One such intervention is based on the existence of the mirror neuron system (MNS).

The MNS consists of several brain areas that become activated while performing an action, as well as while watching someone performing that action [31]. Areas of the MNS are parts of the premotor cortex and parietal lobe, the posterior inferior frontal gyrus, the posterior part of the middle temporal gyrus, and the cerebellum [30]. Because the MNS becomes activated both in action observation and in action execution, it might be expected that observing an action may have a facilitating effect on the execution of that same action. Recent studies have indeed shown that action observation (i.e., a combination of observation and execution of an action), which likely activates the MNS, may have beneficial effects in several patient groups. For example, beneficial effects were found on motor function rehabilitation in people who had had a stroke [32], and on the independence of daily activities in people with Parkinson’s disease [33]. Action observation seems therefore a promising way to positively influence the execution of a function, possibly including the masticatory function.

In this study, we examine whether watching videos of other people chewing has a beneficial effect on the chewing ability of older adults with dementia. The observation of the masticatory movements is added to the execution of that same movement, by showing the videos during lunchtime (i.e., the midday meal). The primary hypothesis is that the observation of people chewing while having lunch will slow down the decline in, or even improve, masticatory ability in older adults with dementia. The secondary hypotheses are that this action observation will have beneficial effects on food intake, cognition, ADL, depression, and QoL.

## Methods/Design

### Study design

This study is an adjusted parallel randomized controlled trial. Data are hierarchically ordered with participants (level 2) and four moments of measurement per participant (level 1). Randomization is being performed at level 2, via a slightly adjusted method due to the setting. Participants are randomly assigned to the experimental or control condition. However, if participants are able to watch each other’s videos, for example, because they sit next to each other during lunch, they are assigned to the same study condition. This is necessary because participants should not be able to watch the videos of the other study condition, in order to have only one study condition per participant. Additionally, an approximately evenly divided number of participants per condition is taken into account per defined group that starts at the same moment (i.e., a nursing home address). Randomization is performed by the researcher, and is done by means of drawing equally sized, folded, pieces of paper. This way the drawing process is performed blindly.

The CONSORT guidelines [34] are followed as strict as possible when reporting the study design, with exception of those for the sections ‘Results’ and 'Discussion’. This exception is due to the type of paper (i.e., a study protocol paper). Furthermore, parts of the method and materials used are the same as, or similar to, those described elsewhere, and some of the tests are there described in more detail [35].

### Setting

The settings where this study takes place are nursing homes or other types of residential care facilities. For the intervention, it is important that these facilities have areas where residents eat together during lunchtime.

### Participants

Participants of this study are older adults (age ≥ 70 years), diagnosed with dementia (as stated in the medical file of a resident). They should have at least mild cognitive decline, which is indicated by a Mini-Mental State Examination (MMSE) [36] score on the Dutch version of the MMSE of ≤ 25. This criterion changed from the criterion in the original study design, which was an MMSE score of 15–25, in order to be able to include a larger group of participants. In addition to the other inclusion criteria, participants should eat with other residents during lunchtime. The latter criterion is decided upon for research purposes (i.e., carrying out the intervention in a more controlled environment), as well as for practical reasons (i.e., the intervention can be carried out easier, and observations can be made in a less intrusive way). Residents are excluded from participation if they have a history of alcoholism or depression, or if they have dysphagia, hydrocephalus, neoplasm, cerebral trauma, personality disorders (other than those related to dementia), disturbances of consciousness, or visual impairments. Visual impairments are a reason for exclusion only if these impairments are present to such a degree that the videos being displayed or the visual stimuli during the test administrations cannot be sufficiently seen by the resident. Residents’ hearing should also be sufficient, that is, spoken communication should be possible.

For the included residents several confounding variables are registered if these are available: age, gender, educational level (by means of the Verhage system [37]), dementia subtype, comorbidities, medication use, use of visual or hearing aids, and use of dental prostheses.

### Materials

#### Masticatory ability

To measure masticatory ability, the two-colour chewing gum test is administered [38]. In this test, the participant receives a piece of chewing gum, consisting of two different colours: blue and pink. This gum is prepared from Bubblicious Cotton Candy (Mondelēz Global LLC, East Hannover, NJ) and Bubblicious Strawberry Splash (Cadbury UK, Birmingham, UK). The test administrator presents the chewing gum with the pink side towards the participant, and asks the participant to chew this piece of chewing gum for 20 s and to return the chewed gum after these 20 s. The chewed gum is then flattened [39] and stored in a sandwich bag for later analysis.

For the analysis of the chewed gums, both sides of the flattened piece of chewing gum are photographed, using predetermined material (i.e., the same digital camera and lights each time) [39] in a predetermined set-up. By means of Mathematica 9 (Wolfram, Oxfordshire, UK) it is analyzed to what extent the two colours are mixed. A more detailed description of this analysis and an explanation of the algorithm can be found elsewhere [39]. The outcome of this analysis is a variable called ‘DiffPix’, which gives an indication of the participant’s mixing ability. Scores range from 0 to 1, in which high values are indicative of little mixing, and low values are indicative of much mixing [39].

#### Food intake

To measure the food intake of the participants, their food is weighed before and after lunch for a period of 5 days. Following the weighed inventory method [40], all prepared ingredients of a participant’s lunch are weighed before lunchtime and the leftovers are weighed after lunch, a procedure described by Fehily and by Marr (as cited in [40]). In the current study, the ingredients are measured separately where possible. If this is not possible, for example, because the ingredients are already put together on the plate, the ingredients are weighed together, and an estimation is made of the ratios of the different ingredients. For situations where it is insufficiently feasible to weigh food (e.g., when participants make their own sandwiches from ingredients placed on the table), the protocol is adapted by the researchers. In these situations, observations are made and specific ingredients that are eaten (including brands where possible) are written down for later calculations. This procedure is largely similar to the direct observation method [40]. After the weighing or observation of food, food intake is calculated in grams and kcals. The latter is being calculated by means of a food composition table, both for the weighing procedure, as mentioned by Adelson and by Widdowson (as cited in [40]), and for the observation procedure [40].

#### Cognition

To measure cognitive function, a neuropsychological test administration takes place. One of these tests, the MMSE [36], is administered as a test to measure general cognition (maximum score: 30). For participants with an MMSE score < 15, only the MMSE is administered as a measure of cognition. This is decided upon in order to still examine changes in cognitive functioning over time, while minimizing the required effort for these participants. For participants with an MMSE score of 15–25, also the following neuropsychological tests are administered.

For the assessment of memory, several tests for short-term memory and long-term memory are administered. For short-term memory, the immediate recall condition of the Eight words test, subtest of the Amsterdam Dementia Screening test [41] is administered (maximum score: 40). In addition, the forward condition of the Digit Span test, subtest of the Wechsler Adult Intelligence Scale III [42] (maximum score: 21 [43]), and the forward condition of the Dutch version of the Visual Memory Span, subtest of the Wechsler Memory Scale [44] (maximum score: 14) are administered. For long-term memory, the delayed recall (maximum score: 8) and recognition (maximum score: 16) subtests of the Eight words test are administered. Additionally, two tests of the Rivermead Behavioural Memory Test [45] are administered: Face recognition and Picture recognition. For Face recognition, possible scores range from −10 to 10, and for Picture recognition, possible scores range from −20 to 20.

Besides memory, also other cognitive domains are assessed. One of these domains is visual integration, for which Picture completion, subtest of the Groninger Intelligence Test [46] is administered (maximum score: 20). For the executive function fluency, the Letterfluency test [47] and two subtests of the Groninger Intelligence Test are administered: Category fluency I (animals) and Category fluency II (professions). Finally, for the executive function working memory, the backward condition of the Digit Span test (maximum score: 21) and the backward condition of the Visual Memory Span (maximum score: 14) are administered.

#### Activities of daily living

The Katz ADL (Dutch version) is used [48] for the assessment of ADL. With this questionnaire, questions on participants’ (in)dependence on ADL are asked of caregivers. The higher the score on this questionnaire, the more dependent a participant is (maximum score: 18).

#### Depressive symptoms

The Cornell Scale for Depression in Dementia [49] is administered to assess depressive symptoms. This questionnaire is filled out by a caregiver [50], and consists of questions on several aspects of depression: mood related signs, behavioral disturbance, physical signs, cyclic functions, and ideational disturbance. A higher overall score on this test is indicative of more depressive symptoms (maximum score: 38).

#### Quality of life

Two questionnaires are completed for the assessment of QoL. The first questionnaire, the Dementia Quality of Life (DQoL) instrument [51], Dutch version [52], is administered together with the neuropsychological test(s). This questionnaire is only administered completely if at least two of three screening questions are answered correctly. The questionnaire contains questions on several aspects of QoL, being self-esteem, positive affect/humor, negative affect, feelings of belonging, and sense of aesthetics. Additionally, there is one question concerning a person’s overall QoL. For all subscales with the exception of the subscale negative affect, a higher score is indicative of a higher QoL. The subscales have different maximum scores [53].

The second QoL questionnaire used is the QUALIDEM [54]. A caregiver fills out this questionnaire, that contains questions on the following aspects of QoL: care relationship, positive affect, negative affect, restless tense behavior, positive self-image, social relations, social isolation, feeling at home, and having something to do. For all these subscales, higher scores are indicative of a higher QoL, and the subscales have different maximum scores.

### Procedure

**Fig. 1:**
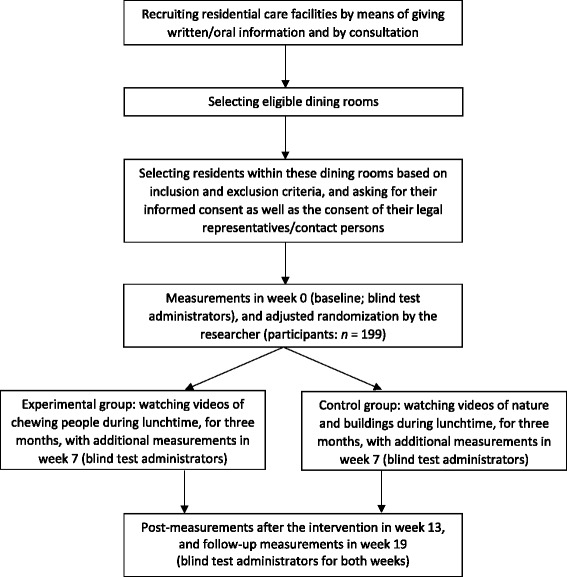
Overview study protocol. Adapted from “The effects of observation of walking in a living room environment, on physical, cognitive, and quality of life related outcomes in older adults with dementia: a study protocol of a randomized controlled trial,” by J.G. Douma, K.M. Volkers, J.P. Vuijk, M.H. Sonneveld, R.H.M. Goossens, and E.J.A. Scherder, 2015, *BMC Geriatrics*, *15*, p. 26. Copyright 2015 by the authors

The study protocol is shown in Fig. 1.

#### Inclusion participants

Residents are selected with regard to the inclusion and exclusion criteria, based on the medical files, by consultation between researcher and medical or nursing staff. The selected residents and their legal representatives/contact persons are asked for their voluntary verbal and/or written consent, and, following this, the MMSE is administered. This test administration is done pre-baseline, in order to examine if the score on the MMSE is ≤ 25 and the participants’ vision and hearing are well enough based on this test administration. If also these inclusion criteria are met, written informed consent is asked from both the resident and his/her contact person, if only verbal consent was given before. When a minimum of two residents and their contact persons provide consent, the intervention can take place in the residential care facility concerned.

#### Interventions

In this study, the experimental condition consists of videos showing people over 50 years of age who are chewing (one person per video), and the control condition consists of videos of nature and buildings which are not expected to activate the mirror neuron system. All videos are without sound, and are shown on tablet-PCs during lunchtime, in settings where residents eat together. A tablet-PC (Samsung Galaxy Tab 2 10.1 or Tab 3 10.1) is placed behind the plate of each participant by a caregiver or volunteer who assists with the lunch. Verbal and written instructions are given on the first intervention day. The researchers may provide additional instructions where requested or deemed necessary. The caregiver or volunteer switches the videos on at the start of the lunch, and off at the end of the lunch. Participants are not specifically asked to pay attention to the videos; they may decide themselves to watch the videos or not.

The videos are shown on weekdays (i.e., Monday to Friday) for 3 months. A period of 3 months is one of the recommendations for physical interventions [55]. All videos have a duration of approximately 40 min, which is a sufficient duration for the lunchtime. Videos are shown during lunchtime, and may therefore be shown shorter than these 40 min if the participant finishes lunch earlier. There are 10 different videos per condition, which means that the participants can watch different videos for 2 weeks, after which the sequence of videos starts over again.

#### Moments of measurement

**Fig. 2:**
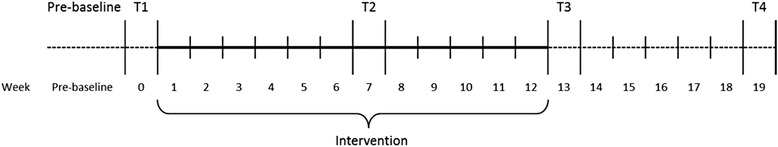
Overview of the moments of measurement and the intervention. Reprinted from “The effects of observation of walking in a living room environment, on physical, cognitive, and quality of life related outcomes in older adults with dementia: a study protocol of a randomized controlled trial,” by J.G. Douma, K.M. Volkers, J.P. Vuijk, M.H. Sonneveld, R.H.M. Goossens, and E.J.A. Scherder, 2015, *BMC Geriatrics*, *15*, p. 26. Copyright 2015 by the authors

To examine the effects of the intervention on masticatory ability, food intake, cognition, ADL, depression, and QoL, there are four moments of measurement. All moments of measurement are planned to take place in the time span of a week. The first moment of measurement is before the start of the intervention (baseline; T1), the second after the first 6 weeks of the intervention (week 7 of the intervention; T2), the third after the 3 intervention months (T3), and the fourth 6 weeks after T3 (T4). See Fig. 2 for a schematic.

During these four moments of measurement, tests and measurements described under the heading ‘Materials’ are administered. One exception is that the MMSE is not administered at T1, as it is administered just before that time as part of the inclusion procedure (pre-baseline). The score on the pre-baseline administration of the MMSE is used as the score at T1. The score on the MMSE per moment of measurement determines whether a complete or shortened test battery is administered. See information under the heading ‘Cognition’, and Table 1 for an overview of the test administrations for participants with different MMSE scores.

**Table 1:** Measurements and test administrations at T1, T2, T3, and T4 for participants and caregivers

	Participant		Caregiver
Measures	MMSE 15-25	MMSE <15	All participants
Primary			
*Masticatory ability*			
Two-colour chewing gum	X	X	
Secondary			
*Food intake*			
Weighed inventory method	X	X	
*Cognition*			
MMSE	X^a^	X^a^	
Eight words test	X		
Digit Span	X		
Visual Memory Span	X		
Face recognition	X		
Picture recognition	X		
Picture completion	X		
Letter fluency	X		
Category fluency I (animals)	X		
Category fluency II (professions)	X		
*ADL*			
Katz ADL			X
*Depression*			
Cornell Scale for Depression in Dementia			X
*QoL*			
DQoL	X	X	
QUALIDEM			X

*MMSE* mini-mental state examination, *ADL* activities of daily living, *QoL* quality of life, *DQoL* dementia quality of life. Adapted from “The effects of observation of walking in a living room environment, on physical, cognitive, and quality of life related outcomes in older adults with dementia: a study protocol of a randomized controlled trial,” by J.G. Douma, K.M. Volkers, J.P. Vuijk, M.H. Sonneveld, R.H.M. Goossens, and E.J.A. Scherder, 2015, *BMC Geriatrics*, *15*, p. 26. Copyright 2015 by the authors

^a^Pre-baseline test score is considered T1 test score

The two-colour chewing gum tests, the neuropsychological tests, and the DQoL are administered face-to-face by test administrators blind to the group allocation of the participant concerned. For the assessment of the neuropsychological tests, test administrators first follow a training. The test administrators are also instructed to pay attention to fatigue of the participants; they can take a break from the test administration if needed. The administration of the chewing gum test is expected to be of low risk, as people with dysphagia are excluded from participation.

The measurements of food intake are predominantly performed by researchers, and in some cases by people working in the care facilities. The questionnaires for the caregivers (i.e., the Katz ADL, QUALIDEM, and Cornell Scale for Depression in Dementia) are handed out at the start of a week of measurements, and are collected at the end of the week. This way, caregivers are able to fill out the questionnaires at a time that fits into their schedules.

#### Treatment exposure

Caregivers are asked to note down per intervention day whether the tablet-PC is placed before each participant. This information is asked for to later control for missed intervention days due to, for example, illness of a resident, or an activity that makes it unfeasible to place the tablet-PCs. In addition, caregivers are asked to note down anything of interest about the residents or the intervention.

To estimate how long the participants watch the videos, observations are made by the researchers. During the course of the intervention, each participant is observed twice. The first observation for a participant takes place in the period between T1 and T2, and the second observation in the period between T2 and T3. An observation takes place from the start to the end of the lunch. Every time the participant watches the video (which is defined by having his/her eyes directed towards the tablet-PC), the duration of watching is noted. In addition, the duration of the lunch is noted down, as well as anything noteworthy being observed. Per observation, the total time the participant watched the video is calculated, and the mean time of the two observation occasions is used as an estimation of the time watched per intervention day.

### Statistical analysis

To examine the difference between the experimental and control group regarding the participant characteristics age, number of comorbidities, number of medicines, and education level, independent sample *t*-tests or Mann–Whitney *U* tests will be used, depending on the level of measurement and distribution of the variables. To compare the two groups on gender, use of aids (i.e., visual aids, hearing aids, and dentures), presence of (types of) comorbidities, and use of (types of) medication, chi-square tests will be used. If participant characteristics differ significantly between groups, these characteristics are included as covariates in the hierarchical mixed model analyses.

Besides the differences in participant characteristics, it will also be examined if groups differ on the baseline scores on the outcome measures (e.g., mastication, or working memory). This is done to examine equality of the experimental and control group before the start of the intervention. These group differences will be analyzed by using independent sample *t*-tests or Mann–Whitney *U* tests; this is dependent on the distribution of the variables.

To examine the effect of the intervention on primary and secondary outcome measures, hierarchical mixed model analyses will be performed. For the primary outcome measure, masticatory ability, mixed models will be fitted with moments of measurement (level 1) nested within participants (level 2). The independent variables are moment of measurement (four levels; baseline as reference category) and group (two levels: experimental condition and control condition). A significant interaction effect of time x group is indicative of an effect of the intervention.

For the secondary outcome measures food intake, ADL, depression, and QoL, the latter being measured by two questionnaires, mixed models will also be fitted. The levels and independent variables are the same as for the primary outcome measure (i.e., masticatory ability). A significant interaction effect of time x group is indicative of an effect of the intervention.

For the remaining secondary outcome measure, cognition, first *z*-scores are calculated for the scores on the cognitive tests. To examine whether specific cognitive domains can be made, factor analyses will be performed using the *z*-scores. Cronbach’s alpha α = .70 will be regarded sufficient to create a cognitive domain [56]. Mixed models will be fitted for the cognitive domains (if they are formed), and for the separate neuropsychological tests. For the separate tests, the raw scores will be used. Again, the same levels and independent variables apply as for the primary outcome measure (i.e., masticatory ability). A significant interaction effect of time x group is indicative of an effect of the intervention.

For all analyses, the statistical software PC-program SPSS will be used. The analyses will be performed using the intention-to-treat method. A significance level of α = .05 applies; however, a Bonferroni correction will be applied where multiple comparisons are made within a domain. The main analyses are tested two-sided.

### Power calculation

Using G*Power 3.1.4 [57], a power analysis was performed for a 2 × 4 mixed factorial design with two groups and four moments of measurement. Setting α at .05, β at .80, and effect size f(V) at .25, the analysis resulted in *n* = 179 participants. When a possible dropout of 10 % was taken into account, this resulted in *n* = 199 participants.

### Ethical considerations

The Medical Ethical Committee of the VU University Medical Center decided this study did not fall within the scope of the Medical Research (Human Subjects) Act (WMO). Further approval was therefore not necessary.

## Discussion

This study aims to examine the effects of video observation of chewing during lunchtime, on masticatory ability, food intake, cognitive function, ADL, depression, and QoL in older adults with dementia. This is being done by showing videos of people chewing (experimental condition) or videos of nature and buildings (control condition) on tablet-PCs during lunchtime in residential care facilities, and comparing the change in the outcome measures (e.g., masticatory ability and cognition) over time between the two groups. Participants in this study observe the chewing movement during the execution of that same movement, that is, while eating. Several studies found that adding observation of a movement to the execution of the same movement (i.e., action observation), improved the performance of the movement more than executing the movement without observing it [32, 33]. Therefore, the current study design is expected be effective for beneficial effects on masticatory functioning to occur. Moreover, the intervention can be easily implemented into residential care, since it consists of only a minimal change in the regular daily setting. In addition, the intervention requires only limited time and effort of caregivers (e.g., placing the tablet-PCs in front of the participants during lunchtime).

The intervention currently takes place in settings where residents eat together. It may be argued that these residents are already being stimulated by the other residents sitting at their table. However, as has been shown in previous studies, masticatory functions change as people get older [5], and many of the residents in nursing homes have low food intake [28]. In contrast, the videos display people who can still chew quite well, and, additionally, the focus of each video is on the face of the person being displayed. Therefore, the videos are still expected to have added value in stimulating the residents in their chewing behavior. In addition, this setting (i.e., a setting where residents eat together) is chosen because it is a relatively controlled environment, and both the intervention and observations are easier to carry out in a shared dining room than in peoples own rooms. Nevertheless, it is important to keep in mind this limitation of the study, that is, the already existing stimulation due to eating together with other residents. However, if beneficial effects are found in this setting, they might be even more pronounced in settings where people eat alone. Possible differences in the effects of watching videos in these different settings may be worth examining at a later stage.

Another limitation of this study could be that the treatment exposure time may be shorter than the intervention time, since participants may choose themselves if they watch the videos or not. This could potentially lead to a shorter actual duration of the intervention. However, it is expected that people watch the videos at least for some time during lunch. In addition, since observations are made in order to estimate how long the participants watch the videos, the treatment exposure time can be taken into account when analyzing the effects of the intervention.

In sum, the goal of this study is to provide insight into possible effects of observation of chewing on mastication itself, but also on food intake, cognitive function, ADL, depression, and QoL of older adults with dementia. This is examined using an intervention that may be easily implemented into daily care if beneficial effects are found. As well as adding to the theoretical knowledge of the effects of action observation in a daily setting on chewing, this intervention may add to the lives of the increasing number of older adults with dementia by slowing down the decline in, or possibly improve, multiple daily life functions.

## Abbreviations

ADL: activities of daily living
DQoL: dementia quality of life
MMSE: mini-mental state examination
MNS: mirror neuron system
QoL: quality of life
